# Synthesis, characterization, and interactions of single-walled carbon nanotubes modified with doxorubicin with Langmuir–Blodgett biomimetic membranes

**DOI:** 10.1007/s11051-018-4239-x

**Published:** 2018-05-12

**Authors:** Dorota Matyszewska, Ewelina Napora, Kamila Żelechowska, Jan F. Biernat, Renata Bilewicz

**Affiliations:** 1 Faculty of Chemistry, Biological and Chemical Research Centre, University of Warsaw, ul. Żwirki i Wigury 101, 02-089 Warsaw, Poland; 2 Faculty of Chemistry, University of Warsaw, ul. Pasteura 1, 02-093 Warsaw, Poland; 3 Faculty of Applied Physics and Mathematics, Gdansk University of Technology, ul. Narutowicza 11/12, 80-233 Gdansk, Poland; 4 Chemical Faculty, Gdansk University of Technology, ul. Narutowicza 11/12, 80-233 Gdansk, Poland

**Keywords:** 1,2-dipalmitoyl-*sn*-glycero-3-phosphothioethanol (DPPTE), Doxorubicin (DOx), Single-walled carbon nanotubes (SWCNTs), Drug carriers, Model lipid membranes

## Abstract

The synthesis, characterization, and the influence of single-walled carbon nanotubes (SWCNTs) modified with an anticancer drug doxorubicin (DOx) on the properties of model biological membrane as well as the comparison of the two modes of modification has been presented. The drug was covalently attached to the nanotubes either preferentially on the sides or at the ends of the nanotubes by the formation of hydrazone bond. The efficiency of the modification was proved by the results of FTIR, Raman, and thermogravimetric analysis. In order to characterize the influence of SWCNT-DOx conjugates on model biological membranes, Langmuir technique has been employed. The mixed monolayers composed of 1,2-dipalmitoyl-*sn*-glycero-3-phosphothioethanol (DPPTE) and SWCNT-DOx with different weight ratio have been prepared. It has been shown that changes in the isotherm characteristics depend on the SWCNTs content. While smaller amounts of SWCNTs do not exert significant differences, the introduction of the prevailing content of the nanotubes increases area per molecule and decreases the maximum value of compression modulus, leading to more fluid monolayer. However, upon increasing the surface pressure, the aggregation of carbon nanotubes within the thiolipid matrix has been observed. Mixed layers of DPPTE/SWCNT-DOx were also transferred onto gold electrodes by means of LB method. Cyclic voltammetry showed that SWCNT-DOx conjugates remain adsorbed at the electrode surface and are stable in time. Additionally, higher values of peak current and DOx surface concentration obtained for side modification prove that side modification allows for more efficient conjugation of the drug to carbon nanotubes.

**Electronic supplementary material:**

The online version of this article (10.1007/s11051-018-4239-x) contains supplementary material, which is available to authorized users.

**Graphical abstract:**
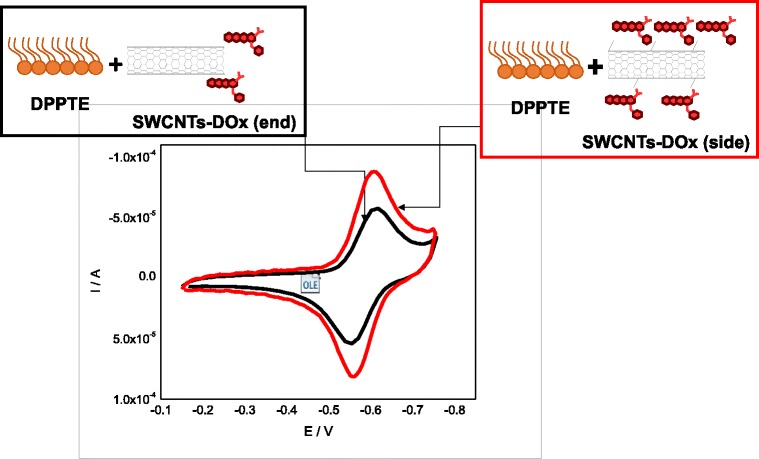
ᅟ

## Introduction

Cancer therapy involves the application of drugs such as doxorubicin (DOx), which is often used in the treatment of leukemia, breast cancer, ovarian cancer, lung carcinoma, and several sarcomas (Cortes-Funes and Coronado 2007). However, due to DOx toxicity, caused mainly by reactive oxygen species and hydroxyl radicals generated in the presence of naturally occurring free iron cations in the Fenton reaction (Minotti et al. 2004), the anticancer treatment with doxorubicin may lead to numerous serious side effects such as drug-induced heart failure. Therefore, various types of drug carriers including liposomes, dendrimers, or cubic phases are being investigated in order to improve the targeted drug transport and reduce the side effects (Li et al. 2014; Ma and Mumper 2013; Nazaruk et al. 2014; Nieciecka et al. 2013).

Single-walled carbon nanotubes (SWCNTs) are one of the potential drug delivery systems, which draw increasing attention mostly due to their properties such as stability, robustness, high drug carrying capacity, and ability to penetrate cell membranes (Peretz and Regev 2012). Although their toxicity raises concern, it strongly depends on the size and functionalization of nanotubes and therefore may be controlled (Yang et al. 2012). The two widely used methods to attach drugs to nanotubes include covalent bonding (Gu et al. 2011) or adsorption to the sidewalls via π-π stacking interaction between the nanotubes and drugs (Liu et al. 2009; Ali-Boucetta et al. 2008). The latter method has been mostly used for the modification of carbon nanotubes with doxorubicin (Liu et al. 2007; Zhang et al. 2009). There is a limited number of reports, in which doxorubicin was attached by forming labile, pH-dependent covalent bond, although it has been proved that SWCNT-DOx conjugates with the covalently bound drug showed improved cytotoxicity compared to the conjugates, in which DOx was adsorbed on the surface of nanotubes by π-π stacking (Gu et al. 2011). The covalent bonding of the drug to a potential drug carrier may be performed by the formation of the hydrazone bond. Stability of this type of bond is pH-dependent: it is stable at pH 7.4 corresponding to healthy cells, but it may be easily cleaved at pH 5.4, which is typical of cancer tissues. Therefore, hydrazone bond has been frequently employed to conjugate DOx to different drug carriers including gold nanoparticles, micelles, or block copolymers (Aryal et al. 2009; Lee et al. 2015; Prabaharan et al. 2009). However, there are only a few examples of doxorubicin attachment to carbon nanotubes via this type of pH-sensitive bonding (Ciobotaru et al. 2014; Fan et al. 2013; Gu et al. 2011; Le et al. 2017). In this paper, the structures and modification procedures are radically different to the ones described in the literature (Fan et al. 2013; Gu et al. 2011). In previous reports, the hydrazone is formed using a long spacer linked to the carboxylic group on the nanotube by the amide bond. The spacer itself ends with the carboxylic group, to which the succinic acid residue with the t-butoxycarbonyl derivative of hydrazine is linked. This method is complicated and a number of by-products with different molecular weights may be formed. On the contrary, the method of functionalization proposed in this paper is much simpler and the probability of the formation of polymer-like by-products is very low. Another long spacer such as poly(styrene-alt-maleic anhydride) was also used by Le et al. (2017) in order to attach doxorubicin via hydrazone bond. The increased drug release at acidic pH due to the pH-sensitive hydrazone bond was proved by UV–Vis experiments. However, also in this case, the method of synthesis is complicated. A much simpler method of covalent attachment of DOx to carbon nanotubes without any linker was also proposed in the literature (Ciobotaru et al. 2014). The covalent bond was formed by carboxyl groups from SWCNTs and amino groups from DOX. The main drawback of such method of functionalization is the lack of pH-sensitivity of the bonding, which results in significantly slower release of the drug from the adduct. Additionally, involving amino groups of DOx in the covalent bonding to carbon nanotubes may decrease the therapeutic effect of the drug, since amino groups in DOx are responsible for its anticancer activity (Beretta and Zunino 2007).

In order to verify the influence of modified carbon nanotubes as drug carriers on the properties of biological membranes the Langmuir technique may be employed. It allows one to form monolayers of amphiphilic substances such as phospholipids, which may be treated as simple models of one leaflet of real biological membranes. This method has been often used to study the interactions of different biologically active species such as drugs, toxins, and proteins with simple models of cell membranes (Dynarowicz-Łątka and Hąc-Wydro 2014; Matyszewska and Bilewicz 2015; Matyszewska et al. 2015). In most cases, these substances are either dissolved in the subphase, on which the membrane is formed and the process of their incorporation into the membrane is monitored or are co-spread with the phospholipids constituting the model layer (Geraldo et al. 2013). As a result, the characteristic features of monolayers such as the area per molecule, collapse pressure, and the organization of the layers change. Additionally, many substances induce the fluidization of the phospholipid layer, which is manifested by the decrease in the compression modulus (Sandrino et al. 2014).

**Fig. 1:**
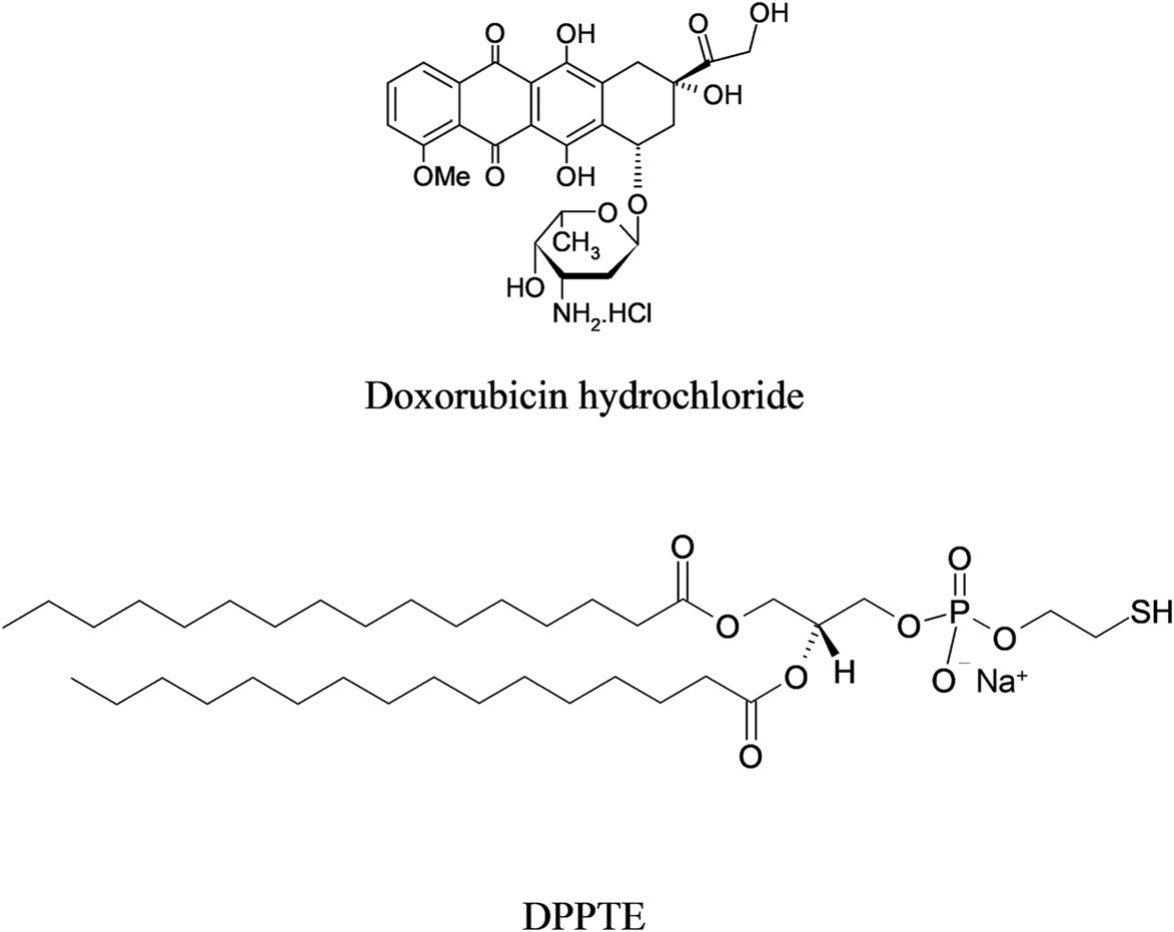
Structures of doxorubicin (DOx) and 1,2-dipalmitoyl-*sn*-glycero-3-phosphothioethanol (DPPTE) used as the component of model membranes

**Fig. 2:**
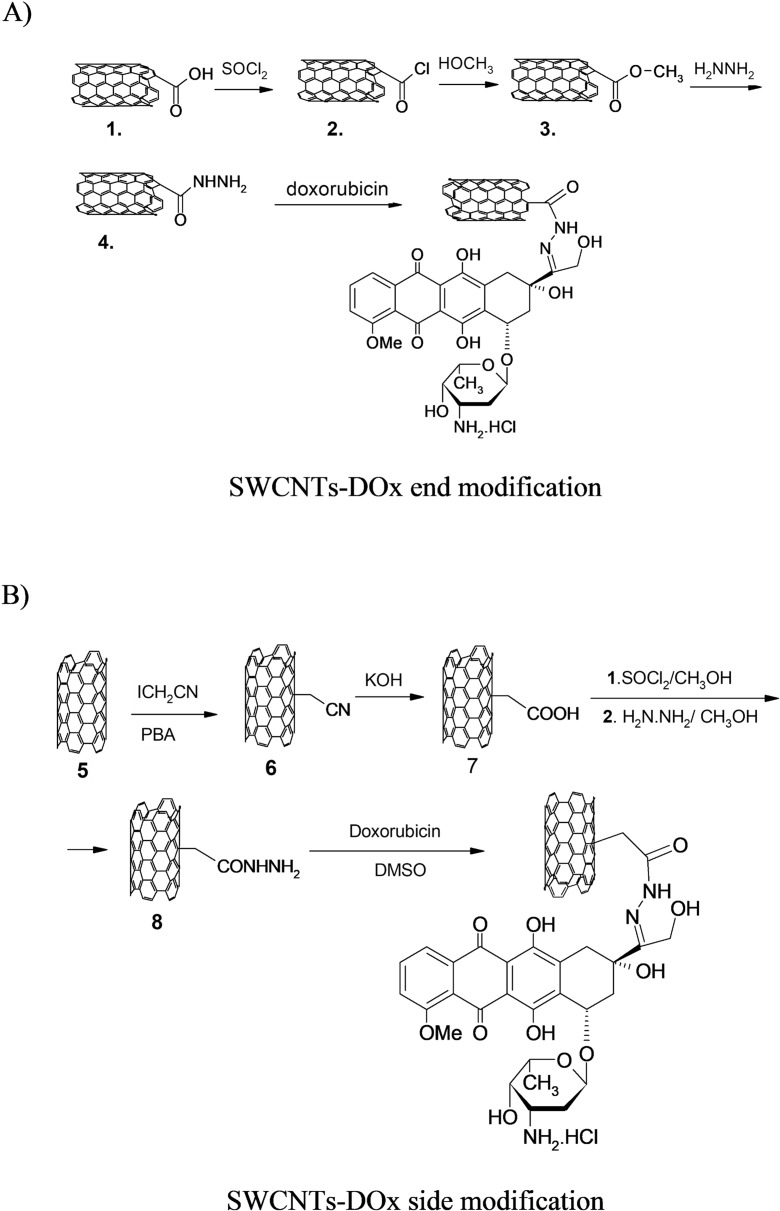
Synthesis of **a** SWCNT-DOx end conjugate and **b** SWCNT-DOx side conjugate

In this paper, we report the synthesis, characterization, and the results of the studies on the influence of carbon nanotubes modified with an anticancer drug doxorubicin (Fig. 1) on the properties of model biological membranes as well as the comparison of the type of the modification of the nanotubes with the drug. Single-walled carbon nanotubes were modified with the anticancer drug preferentially either at the ends or sides of the nanotubes using the covalent bonding via the formation of hydrazone bond (Fig. 2). After the initial characterization of the carbon nanotube–drug adducts by performing TGA and spectroscopic analysis, their interactions with model biological membranes were investigated using Langmuir technique. A thiolipid 1,2-dipalmitoyl-*sn*-glycero-3-phosphothioethanol (DPPTE) (Fig. 1) was employed as a component of biomimetic systems due to its resemblance to 1,2-dipalmitoyl-*sn*-glycero-3-phosphocholine (DPPC)—a phospholipid commonly occurring in natural cell membranes. Additionally, our previous results of Langmuir studies show that anthracycline anticancer drugs (daunorubicin) in a free form may easily incorporate into the DPPTE layers during their formation and significantly change the properties of the layers (Matyszewska and Bilewicz 2015). The interactions of this anticancer drug with model DPPTE membranes were also initially compared for the drug in the free form and attached to the carbon nanotubes showing that the amount of the drug present in the model membrane after interactions is comparable irrespective of its form (free or attached to CNTs) (Matyszewska 2016). However, the influence of the daunorubicin-carbon nanotube adduct on the properties of thiolipid monolayers seemed to be not that significant. Another issue that should be taken into account while using DPPTE as a component of model membranes is the fact that the presence of a thiol group in the head group region of a DPPTE molecule leads to the overall negative charge of the polar head of the lipid. It is important, since the recent scientific reports show that the charge of both carbon nanotubes and the lipids constituting the model membrane may strongly influence the interactions between CNTs and the membranes (Jiang et al. 2017). The authors point out that the opposite charges of a model membrane and carbon nanotubes electrostatically mediate interactions. Additionally, it was observed that MWCNTs with a high defective degree disrupted the positively charged membrane and extracted phospholipids from the negatively charged membranes. Therefore, in the present investigations, 1,2-dipalmitoyl-*sn*-glycero-3-phosphothioethanol (DPPTE) was employed as a component of a model membrane, which allowed us both to refer to our previous results and also to consider the influence of electrostatic interactions between polar heads of lipid and carbon nanotubes.

Langmuir monolayer studies are accompanied by electrochemistry measurements to characterize the supported mixed layers of DPPTE and modified SWCNTs and to compare the two types of carbon nanotubes’ modification where anticancer drugs are attached preferentially at the ends or at the sides of the nanotubes.

## Experimental

### Covalent functionalization of carbon nanotubes

#### End functionalization of SWCNTs

The characteristic of commercial, oxidized SWCNT(s)-COOH (CheapTubes, Brattleboro, USA) used for end functionalization is as follows: diameter 1–2 nm, purity > 90%, ash < 1.5%, length 5–30 μm, specific surface area 407 m^2^/g; the content of –COOH groups of functionalized SWCNT equals to 2.73%. The nanotubes were used as received.

#### Hydrazide of SWCNT-end

Chlorocarbonyl SWCNTs **2** (Fig. 2a) were prepared by treating 50 mg of SWCNT-COOH **1** with 1 ml thionyl chloride and a trace amount of pyridine. The mixture was maintained sonicated at 50 °C for 4 h. Then, the excess of thionyl chloride was removed under reduced pressure. The solid residue was suspended in 3 ml of dry toluene, sonicated for a while, and the solvent was evaporated again under reduced pressure to remove residual thionyl chloride. After two repetitions of the last procedure, the solid material **2** was mixed with 1 ml of anhydrous methanol, sonicated for a while, and left overnight. Then, the solvent was evaporated and the ester **3** was mixed with 1 ml of 90% hydrazine hydrate. After short sonication (5 min), the suspension was left for 2 days at room temperature to form **4** (Hirsch 2002; Khazaei et al. 2010). The solid after exhaustive washing with methanol was dried under reduced pressure.

#### Reaction product of SWCNT-end hydrazide with doxorubicin

This part of synthesis was partly inspired by the method previously described in the literature (Gu et al. 2011), but in this case, the drug is directly linked to SWCNT-CONHNH_2_ hydrazide and not through the pegylated derivative of nanotubes. First, a solution of 20 mg doxorubicin hydrochloride (AK Scientific, USA) in 4 ml dimethylsulfoxide (DMSO) was prepared. Then, 70 mg of material **4** (Fig. 2a) was added to the solution; the mixture was sonicated at room temperature for 2 h and left for 2 days at room temperature in the dark. Then, the suspension was centrifuged. The solid was washed exhaustively with methanol until the filtrate became colorless. The pure SWCNT-DOx end (Fig. 2a) was dried under vacuum.

#### Side functionalization of SWCNTs

Characteristics of pristine SWCNTs received from CheapTubes (Brattleboro, USA) were presented in our previous publication (Sadowska et al. 2009). The mode of the synthesis was inspired by the suggestions by Peng et al. (2003) and Khabashesku and Pulikkathara (2006) and by a protocol previously described for partially preferential synthesis of side and end-carboxylated SWCNTs (Nazaruk et al. 2010).

Cyanomethylation of the starting pristine SWCNTs **5** (Fig. 2b) was performed as described with minor modifications (Nazaruk et al. 2010). The formed side cyanomethylated SWCNTs **6** were hydrolyzed using 5% KOH solution in water-ethanol (1:2) mixture at 70 °C for 10 h. The resulting insoluble carboxylic acid **7** was esterified with an excess of methanol upon addition of equivalent amount of thionyl chloride. After 2 days at room temperature, the solvent was removed under reduced pressure and a tenfold excess of 90% hydrazine hydrate was added to the residue. After 2 days, the hydrazide **8** was collected, washed, and vacuum dried. Its condensation with doxorubicin hydrochloride was performed as in the case of SWCNT-DOx-end.

Thermal analysis was performed on 4 mg of samples in a flowing argon current of 50 cm^3^/min using Netzsch STA 449F1 apparatus. The heating rate was 10°/min in the temperature range from 40 to 900 °C.

IR spectra of the starting CNTs and the final modification products were registered using Frontier FTIR/FIR Perkin Elmer spectrometer. Samples (3% by weight) were ground with dry KBr in agate mortar and pressed to form pellets. Spectra were collected in the MIR range (4000–600 cm^−1^). Twenty scans were averaged per sample. Spectral resolution was 2 cm^−1^.

Raman spectra were recorded using Renishaw InVia spectroscope with argon ion laser operating at 514.5 nm focused through a × 50 objective. Collected light was dispersed through a triple monochromator and detected with a charge-coupled device. The spectra were collected in dark, with resolution of 2 cm^−1^ in the range of 100–3200 cm^−1^.

### Monolayer formation at the air–water interface

1,2-Dipalmitoyl-*sn*-glycero-3-phosphothioethanol (sodium salt) (DPPTE) (Avanti Polar Lipids, USA) was dissolved in chloroform (Avantor) to give 1 mg/ml stock solution. Mixed DPPTE/SWCNT-DOx (end or side modification) dispersion was prepared by weighing 0.5 mg of nanotubes, adding the appropriate volume of DPPTE stock solution, and filling up with chloroform in order to obtain the desired weight ratio of carbon nanotubes to thiolipid. Two different types of the mixed layers were studied: with the prevailing amount of thiolipid and with the prevailing amount of nanotubes. Prior to the deposition at the air–water interface, the mixed solutions were sonicated in the ultrasonic bath (Emag, Germany) for approximately 30 min to ensure carbon nanotube dispersion. Monolayers at the air–solution interface were formed using a Langmuir trough KSV LB 5000 (KSV-Nima, Finland) equipped with two movable hydrophilic barriers and a Wilhelmy balance with a paper Wilhelmy plate (paper plate was changed after each experiment). The trough was controlled by a computer using KSV-5000 software version. Monolayers were prepared on Milli-Q ultra-pure water (resistivity 18.2 MΩ/cm). After cleaning the subphase, an appropriate amount (a few microliters) of either DPPTE or mixed DPPTE/SWCNT-DOx dispersion was spread at the interface. The exact amount of the solution/dispersion was calculated in such a way that the spread molecules behave as a two-dimensional gas, are far apart, occupying the area freely, and do not interact with each other. As a result, the film pressure is therefore undetectable. Next, the solvent was allowed to evaporate for approximately 15 min and the compression of the film was performed at a speed of 10 mm/min (corresponding to the speed of 7.5 cm^2^/min for a trough width of 7.5 cm). Since the compression rate does not influence the shape of the π-A isotherm of most lipids (Jyoti et al. 1996), this parameter has been kept constant. The Langmuir experiments have been performed at constant temperature of 21 ± 1 °C.

### Monolayer deposition on solid support and electrochemical experiments

Supported DPPTE/SWCNT-DOx (1:10 *w*/*w*) monolayers were prepared by Langmuir–Blodgett technique and were deposited on gold electrodes (10 × 10 mm slides, Ssens, The Netherlands), which were 200–300-nm-thick gold films evaporated onto borosilicate glass precoated with an underlayer of chromium. Prior to the deposition, the gold substrates were cleaned in the mixture of H_2_O_2_:NH_3_:H_2_O with 1:1:5 ratio at 70 °C for approximately 10 min, rinsed with Mili-Q water, and flame annealed. The DPPTE/SWCNT-DOx layers were transferred from the air–water interface onto gold electrode at a surface pressure of 35 mN/m. The layers were deposited on the gold surface by vertical withdrawal of the electrode at the speed of 25 mm/min to give a transfer ratio of 1.0 ± 0.1. The modified electrodes were dried in air for 2 h.

The SEM images were taken with Merlin field-emission scanning electron microscopy system (Zeiss, Germany) controlled by the producer’s software. Electrochemical experiments were performed by means of AutoLab AUT 71819 with the GPES 4.9 software in the three-electrode system with Ag/AgCl as a reference electrode and platinum foil (10 × 10 mm plate) as a counter electrode. The supporting electrolyte was 50 mM phosphate buffer, pH = 6.9 (sodium phosphates from Avantor, Poland).

## Results and discussion

### Characterization of modified nanotubes

**Fig. 3:**
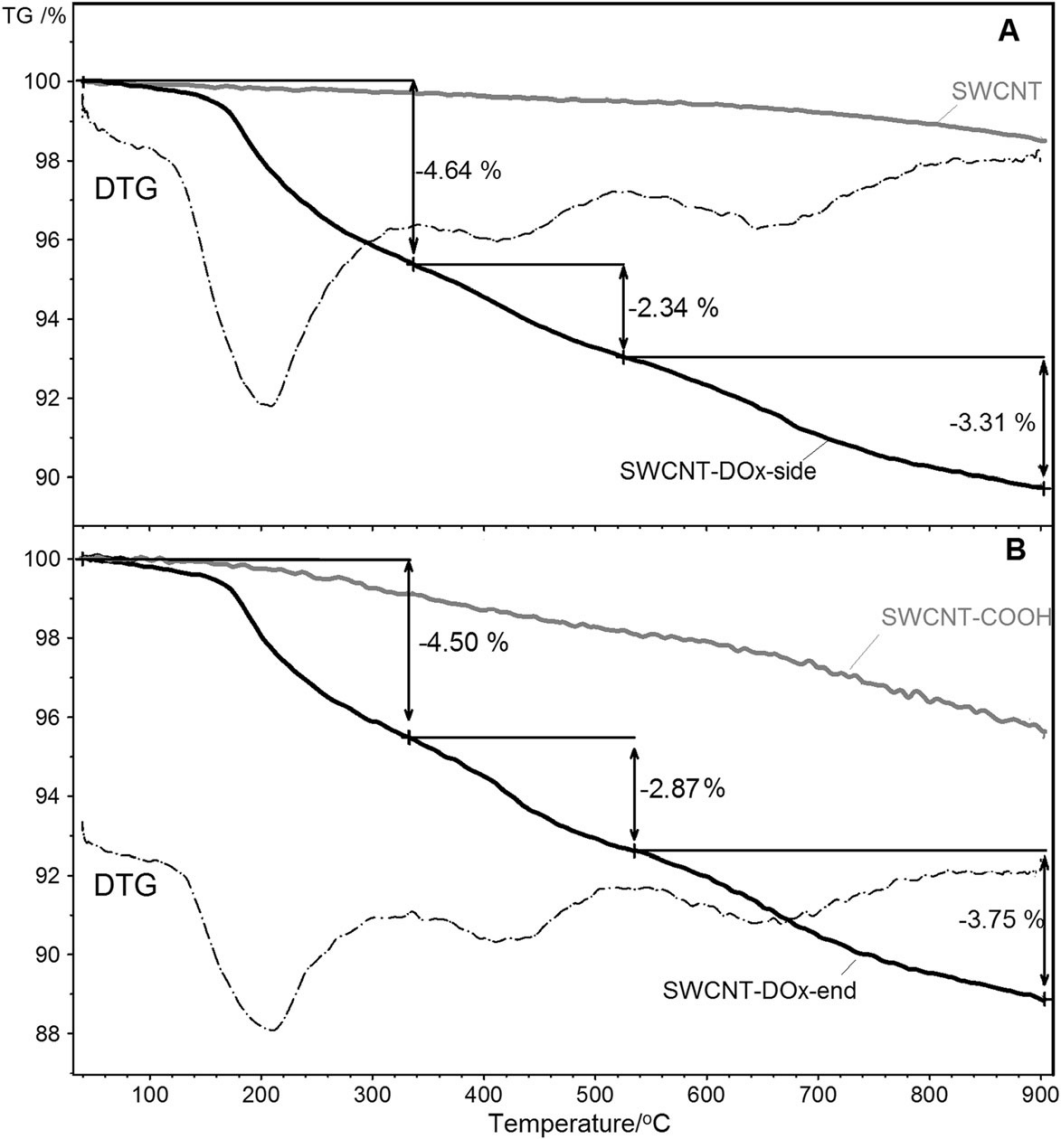
TG and DTG curves for **a** SWCNT-DOx side modification and **b** SWCNT-DOx end modification

The thermogravimetric (TG) analysis was performed to establish the degree of the SWCNT functionalization. Figure 3a shows results of thermogravimetric studies of SWCNT-DOx-side sample with its precursor, which is pristine SWCNT. The TG curve profile for pristine SWCNTs confirmed high purity of the material, as c.a. 1% mass loss was obtained in the whole range of analysis. The total mass loss was higher after functionalization, reaching near 11% for SWCNT-DOx-side sample, and the TG curve profile was substantially different. The first observable mass loss for SWCNT-DOx-side is present below 320 °C with maximum at around 190 °C. In this range, the decomposition of doxorubicin’s sugar moiety may occur (Shafizadeh 1971). The small mass loss observed below 150 °C is equal to ca. 0.5% and can be ascribed to the residual solvent evaporation. The second, more subtle mass loss was registered between 320 and 520 °C, with maximum at ca. 420 °C. The functionalization degree was calculated considering the mass decrease in the recorded temperature range, subtracting mass loss due to solvent evaporation and mass loss observed for pristine nanotubes. The functionalization degree was ca. 1.8·10^−3^ mol of bonded doxorubicin per 1 mol of carbon atoms.

Analogous study performed for SWCNT-DOx-end (Fig. 3b) gave total mass loss equal to approximately 11%. Similarly, disintegration of carbohydrate moiety at around 195 °C, followed by further decomposition of DOx moiety, was observed at ca. 420 °C. The mass loss indicates the functionalization degree equal to 1.5·10^−3^ mol of substituent per 1 mol of carbon. SWCNT-COOH was analyzed as a reference (Fig. 3b, gray line). The total mass decrease for SWCNT-COOH was monotonic and was equal to 4.36%, with no distinct maxima at DTG curve (data not shown).

The thermogravimetric curves are very similar for both DOx functionalized samples, proving successful functionalization with DOx moieties. The functionalization degree for both types of derivatized CNTs was comparable, but the substitution degree in the case of side form is slightly higher. According to the literature, the decomposition of DOx is observed in the temperature range of 175–800 °C; however, most of the DOx does not decompose to vapor state and is collected as a char in the crucible after TG analysis (Manocha and Margaritis 2010). Even heating to 900 °C leads to only c.a. 50% mass loss of doxorubicin. Such behavior increases the generally big error of the calculated functionalization degree of SWCNTs.

**Fig. 4:**
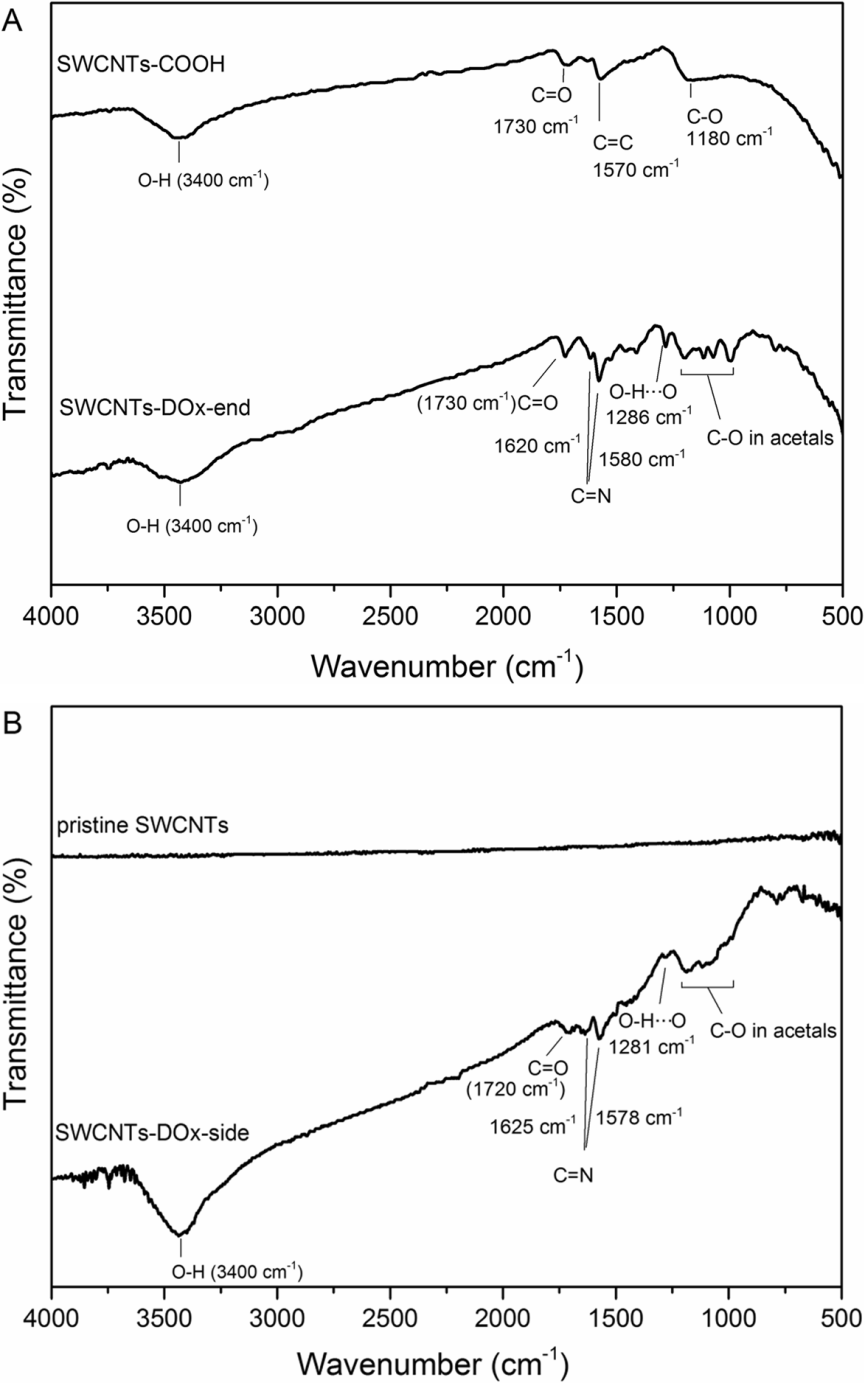
FTIR spectra of doxorubicin functionalized carbon nanotubes with reference to starting material. **a** SWCNT-DOx end conjugate with reference to carboxylated SWCNTs and **b** SWCNT-DOx side conjugate with reference to pristine SWCNTs

FTIR spectroscopy was also used to confirm the chemical functionalization of carbon nanotubes with DOx. In Fig. 4a, the spectrum of SWCNT-DOx-end conjugate with reference to the spectrum of carboxylated SWCNTs is shown. In both spectra, broad bands centered at 3400 cm^−1^ and well-developed bands at 1730 cm^−1^ can be observed. They refer to O–H bonds vibrations and bending of C=O bonds, respectively. In the case of SWCNT-DOx-end sample, the spectrum is more complex and new bands appeared as compared to the starting material. Sharp bands at 1620 and 1580 cm^−1^ are in the region of C=N frequencies, confirming the hydrazone bond formation. They can be also ascribed to DOx aromatic ring stretching and breathing as proposed by Das et al. (2010). Characteristic band for DOx at 1286 cm^−1^ can be also noticed. It originates from bending of O–H···O, that is hydroxyl groups associated with quinone groups by hydrogen bonds in hydroxylanthraquinone ring of DOx. Other bands in the range of 1200–990 cm^−1^ also confirm the presence of DOx molecules in the analyzed sample.

In the case of side-modified nanotubes, the recorded spectrum is completely different from the spectrum of starting material-pristine nanotubes (Fig. 4b). As expected, there are no distinct bands in the pristine nanotube’s spectrum, as they possess mainly carbon-carbon bonds. On the other hand, SWCNT-DOx-side sample revealed spectrum similar to SWCNT-DOx-end spectrum. Broad stretching band referring to O–H bonds present in DOx moiety is observable at 3400 cm^−1^. Similarly, sharp bands at 1625 and 1578 cm^−1^, which are in the region of C=N frequencies, confirm the chemical functionalization of CNTs.

**Fig. 5:**
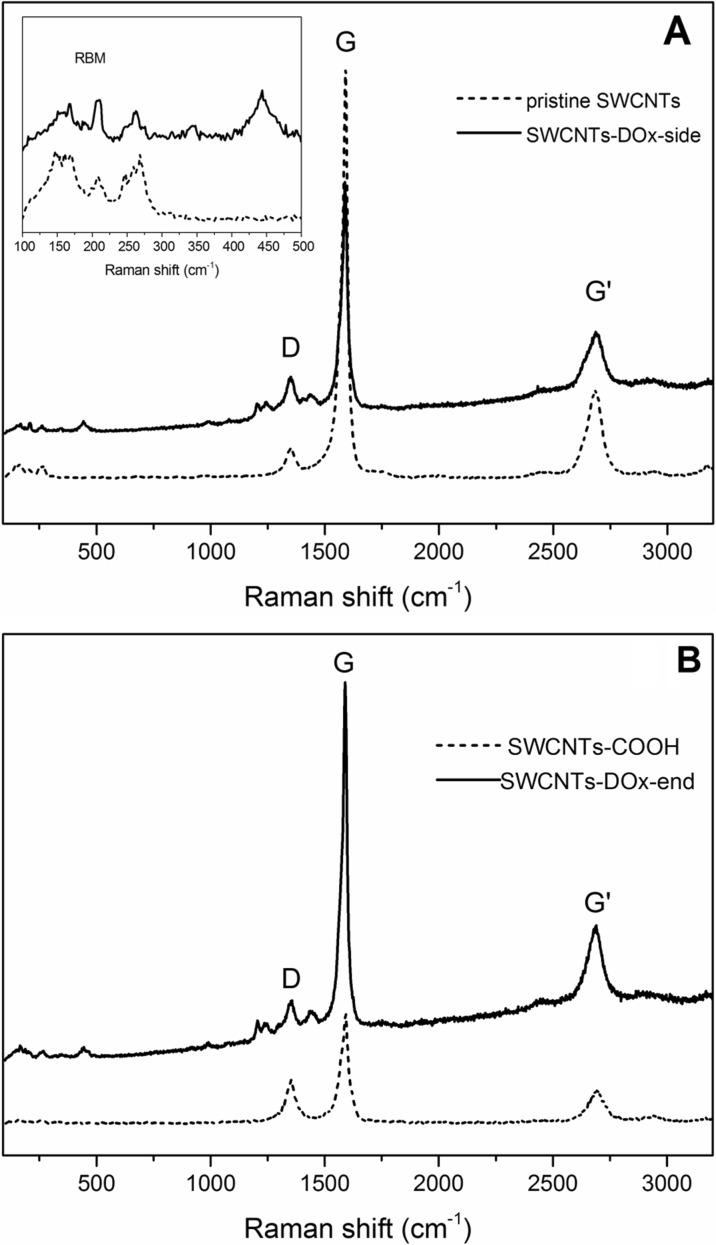
Raman spectra of nanotubes functionalized with doxorubicin: **a** SWCNT-DOx-side functionalized with reference to pristine SWCNTs. Inset: enlarged RBM region for pristine and functionalized nanotubes. **b** SWCNT-DOx-end functionalized with reference to carboxylated SWCNTs

The covalent chemical functionalization of CNTs was also confirmed by Raman spectroscopy. The Raman spectra of pristine and functionalized SWCNT-DOx-side samples are presented in Fig. 5a. Raman spectrum of pristine single-walled carbon nanotubes reveals four bands at ~ 1350 cm^−1^ (D-band), ~ 1590 cm^−1^ (G band), G′ band at ~ 2700 cm^−1^ and RBM band in the range of 100–400 cm^−1^. The ratio of D to G band intensities is used to estimate the quality and/or purity of carbon nanotubes. It is believed that after chemical functionalization, the I_D_/I_G_ ratio should increase as compared to the pristine material because the hexagonal lattice of carbon is disrupted. In addition, more accurate measurement can be achieved by comparing the ratio containing the intensity of G′-band peak that is I_G΄_/I_D_ and I_G΄_/I_G_. Since the G′ band results from a two-phonon coupling, its intensity should decrease as the sample becomes less ordered. It can be seen in Fig. 5a that spectrum of SWCNTs after functionalization differs from spectrum of pristine nanotubes. The comparison of bands’ ratios is given in Table 1. Based on the analysis of the obtained data, it can be concluded that the SWCNT-DOx-side sample was successfully functionalized on the side walls as an increase in I_D_/I_G_ ratio with simultaneous decrease in I_G΄_/I_D_ and I_G΄_/I_G_ can be noticed. Moreover, the radial breathing mode (RBM), which is a feature unique to SWCNT relative to other sp^2^ carbons, is of particular significance. The multi-band RBM mode is sensitive to chemical functionalization and disappears or is diminished after addition of chemical moieties to the nanotubes’ skeleton. It can be seen in Fig. 5a that for SWCNT-DOx-side sample, the RBM mode alignment is different than for the starting material. For pristine SWCNTs, the most intensive bands are present at 148, 159, and 165 cm^−1^ in the low wave number range and at 248, 258, and 268 cm^−1^ in the higher wave number range. After side wall functionalization with DOx moieties, bands at 148, 159, and 248–268 cm^−1^ were reduced but new bands appeared with the most distinct bands at 210 and broad band centered at 440 cm^−1^. These bands correspond to C–C stretching and to C–C–O and C–O in DOx molecule, respectively. Moreover, Raman spectrum of SWCNT-DOx-side revealed other bands lying in the range characteristic for doxorubicin. Additional bands, observed in the range from 1100 to 1300 cm^−1^, can be attributed to C–O, C–O–H, and C–H present in doxorubicin. Bands related to skeletal ring vibrations in DOx molecule can be also found in SWCNT-DOx-side Raman spectrum between 1400 and 1500 cm^−1^.

**Table 1:** The intensity ratios of Raman bands for analyzed samples

Intensities ratio	SWCNT(s)	SWCNT-DOx-side	SWCNT(s)-COOH	SWCNT-DOx-end
I_D_/I_G_	0.06	0.10	0.34	0.06
I_G΄_/I_D_	3.50	2.20	0.18	3.34
I_G΄_/I_G_	0.23	0.20	0.06	0.20

Similarly, SWCNT-DOx-end sample was analyzed using Raman spectroscopy with reference to SWCNT(s)-COOH material. The results are presented in Fig. 5b and in Table 1. In the case of SWCNT-COOH, the I_D_/I_G_ ratio is relatively high and is reduced for the SWCNT-DOx-end sample. It was mentioned in the literature that the D band originates mainly from the amorphous carbon in the sample (even if present only in small amount) and is less responsive to defects in tube walls (Osswald et al. 2005). Here, the decrease of D vs. G band intensity may be due to the removal of amorphous carbon and the most defective tubes during washing after functionalization with DOx moieties. Similar results were documented previously (Sadowska et al. 2010; Long et al. 2008). Characteristic bands for DOx molecule can be also found in the Raman spectrum of SWCNT-DOx-end sample (Fig. 5b). Bands referring to C=C in aliphatic chains and C=O bonds can be observed below 500 cm^−1^. Bonds such as C–O, C–O–H, and C–H present in doxorubicin give rise to the bands present between 1100 and 1300 cm^−1^. Skeletal hydroxylanthraquinone ring vibrations in DOx molecule can be found in SWCNT-DOx-end Raman spectrum in the range of 1400 and 1500 cm^−1^.

### Langmuir studies of the influence of SWCNT-DOx adducts on the properties of DPPTE membranes

In order to study the influence of carbon nanotubes modified with the anticancer drug on the model membranes composed of DPPTE, the Langmuir technique has been employed. Although nanotubes modified with water-soluble DOx exhibit some water solubility, it is not enough to perform studies, in which they could be added into the subphase to observe their incorporation into the monolayer during its formation, which forces the preparation of mixed layers. On the other hand, the ability to form stable monolayers of nanotubes was only reported when the nanotubes were modified with amphiphilic species, which are able to form monolayers on their own (Fu et al. 2012; Jia et al. 2008; Liu et al. 2006). In this case, the anticancer drug doxorubicin employed to modify the CNTs does not form stable monolayers at the air–water interface and therefore does not provide for the film-forming properties of carbon nanotubes. However, it has been previously shown that CNTs, which by themselves do not form monolayers, may still form mixed monolayers with other monolayer-forming species such as octadecanol (Sadowska et al. 2009). Therefore, the mixed layers of thiolipid and carbon nanotubes with covalently attached doxorubicin at the end and sides of the nanotubes were prepared, which allowed for the investigation of the influence of CNTs on model membranes.

**Fig. 6:**
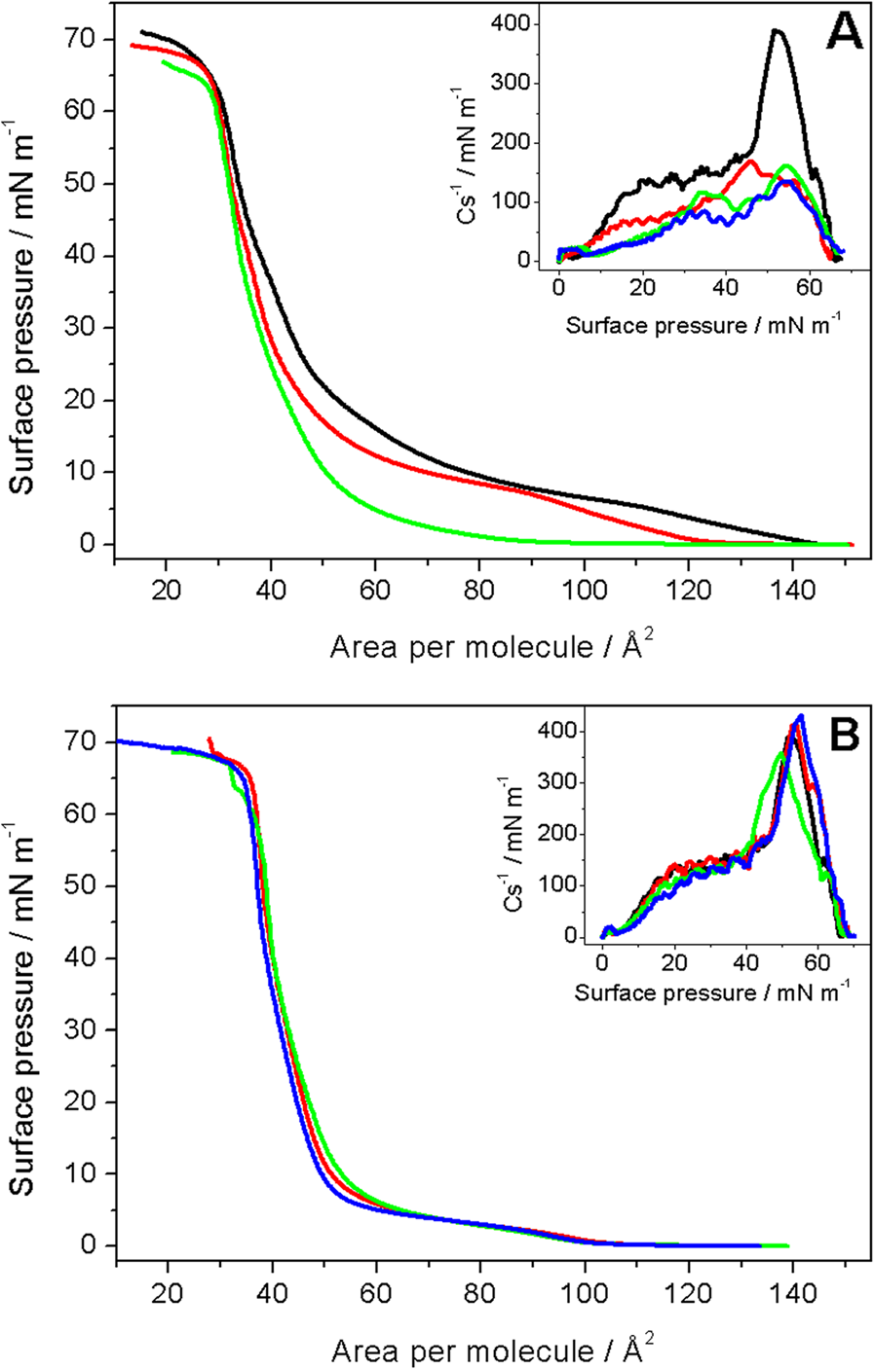
Surface pressure-area per molecule (π–A) isotherms of DPPTE monolayer (black) and mixed layers of DPPTE/SWCNT-DOx (end modification) **a** weight ratio of 1:10 (red), 1:5 (green), 1:3 (blue); **b** weight ratio of 10:1 (red), 5:1 (green), 3:1 (blue) formed on water subphase. Insets: compression modulus vs. surface pressure plots

Results of Langmuir studies show that there is no difference in the surface behavior between the side and end modification of nanotubes. The isotherms obtained in the same experimental conditions for these two types of nanotubes were identical. Therefore, we show only Langmuir data for the end modification. Two types of mixed DPPTE/SWCNT-DOx (end modification) layers were investigated: with the prevailing weight ratio of nanotubes (Fig. 6a) and prevailing weight ratio of thiolipid (Fig. 6b). It can be clearly seen that when the amount of modified nanotubes in the mixed layer is higher than the amount of thiolipid, the characteristic parameters of the layer change compared to monocomponent DPPTE monolayers (Table 2). With the increasing content of CNTs in the mixed layer, the area per molecule in the monolayer increases from the value of approximately 44 Å^2^ for the monocomponent DPPTE monolayer to 47 Å^2^ for the highest investigated ratio of nanotubes. It implies that carbon nanotubes occupy the space designated for the DPPTE molecules in the monolayer at the air–water interface. In the same time, the area corresponding to the collapse of the monolayer decreases in the presence of carbon nanotubes in the monolayer, which results in the slightly different shape of the π-A isotherm compared to monocomponent DPPTE monolayers (Fig. 6a). Similar observation was made by Lo et al. who studied the Langmuir films of multi-walled carbon nanotubes (MWCNTs) co-dispersed in chloroform with poly(3-hexylthiophene) (P3HT) with different weight ratio (Lo et al. 2010). With the increasing ratio of MWCNTs, the isotherms were shifted towards larger areas. Interestingly, the presence of the prevailing amount of nanotubes does not influence the value of collapse pressure. Since the shape of the isotherm in the presence of carbon nanotubes is altered mostly in the range of larger areas per molecules and at lower values of surface pressures, it may be supposed that in this region, the influence of SWCNTs is the most significant. These conclusions are also consistent with the results obtained by Seemork et al. (2016), who showed that carbon nanotubes and other carbon materials bind much easier to the disordered lipid bilayer membrane of liposomes consisting of DOPC as compared to the ordered membrane consisting of DMPC/cholesterol. Upon the further compression of the barriers and the formation of more ordered thiolipid layer, the nanotubes cannot be squeezed out from the monolayer into the subphase because they are not enough water-soluble. However, it is likely that decreasing the area of the air–water interface may lead to the formation of aggregates and bundles of nanotubes, which do not occupy that much interfacial area. As a result, the increase in the area per molecule in the organized monolayer is not as significant even in case of the highest content of the nanotubes in the mixed layer (Table 2).

**Table 2:** Characteristic parameters of monocomponent DPPTE and mixed DPPTE/SWCNT-DOx Langmuir monolayers formed on water subphase

Subphase	A_0_/Å^2^	A_coll_/Å^2^	π_coll_/mN m^−1^	Cs^−1^/mN m^−1^
DPPTE	43.9 ± 0.3	36.6 ± 0.2	65.2 ± 1.7	395 ± 25
Prevailing amount of SWCNTs in the mixed monolayer				
DPPTE/SWCNT-DOx 1:3	44.2 ± 1.5	29.9 ± 1.5	63.3 ± 0.8	140 ± 25
DPPTE/SWCNT-DOx 1:5	46.9 ± 1.6	30.6 ± 1.2	65.7 ± 1.3	160 ± 15
DPPTE/SWCNT-DOx 1:10	47.4 ± 1.5	29.6 ± 1.0	66.2 ± 1.1	170 ± 15
Prevailing amount of DPPTE in the mixed monolayer				
DPPTE/SWCNT-DOx 3:1	43.4 ± 0.7	36.1 ± 0.2	66.9 ± 1.4	430 ± 25
DPPTE/SWCNT-DOx 5:1	44.9 ± 0.2	36.3 ± 0.8	66.0 ± 1.7	360 ± 10
DPPTE/SWCNT-DOx 10:1	44.1 ± 1.0	35.9 ± 0.4	65.2 ± 1.2	375 ± 25

**Fig. 7:**
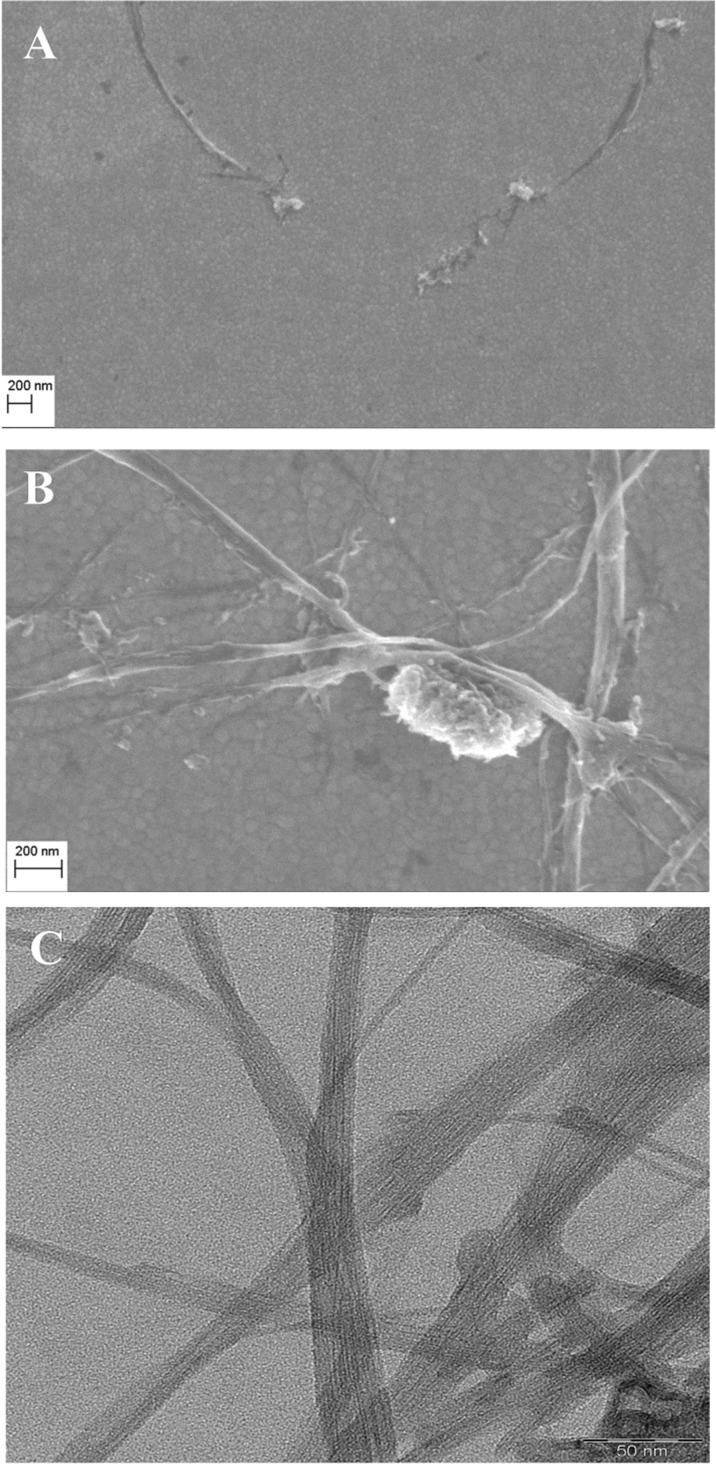
SEM image of DPPTE/SWCNT-DOx (1:10 *w*/*w*, end modification) monolayers transferred by LB method onto gold slides **a** at 10 mN/m and **b** at 35 mN/m. **c** TEM image of SWCNT-DOx (end modification)

**Fig. 8:**
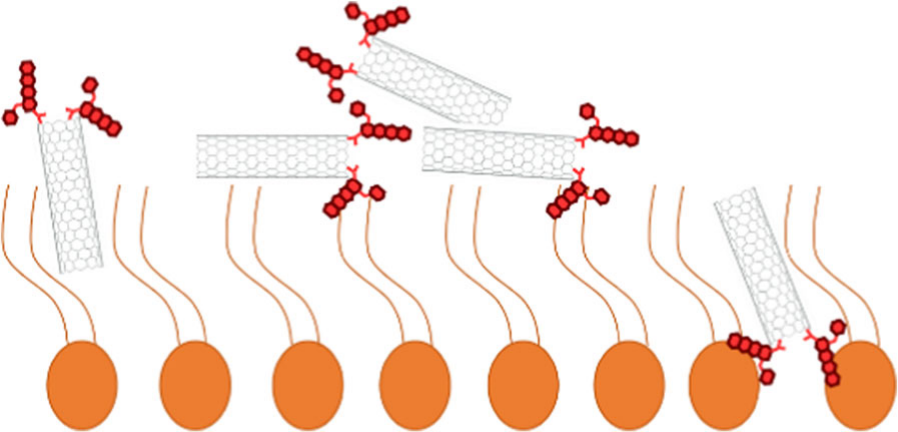
A scheme representing the possible mechanisms of interactions between carbon nanotubes (end modification as an example) and thiolipid monolayer

This observation was also confirmed by electron microscopy. SEM images were taken for supported mixed DPPTE/SWCNT-DOx (1:10 *w*/*w*) layers, and TEM images were taken for pure SWCNT–drug adduct. The resolution of TEM is not sufficient to discriminate between the two types of modification; therefore, Fig. 7c shows the exemplary TEM image of single-walled carbon nanotubes modified with DOx at the end of the nanotubes. The SEM images taken for the mixed layers compressed to 10 mN/m show individual nanotubes on top of the grained-like structure of lipid in the background (Fig. 7a). However, the images obtained for the mixed layers compressed to 35 mN/m and transferred onto gold electrodes prove that carbon nanotubes do not form truly miscible mixed layers with the thiolipid. Due to the interactions between them, carbon nanotubes form aggregates or bundles (Fig. 7b), which may be observed along with individual nanotubes when the layer is compressed to higher surface pressures. It is also interesting to note that nanotubes have the parallel orientation with respect to the thiolipid matrix and electrode surface, which is consistent with the results presented by Lacerda et al. (2013), who studied the interactions of functionalized carbon nanotubes with biological membranes. It has been shown that in case of the interactions of carbon nanotubes with human lung epithelial cells, the nanotubes at first have the parallel orientation with respect to the cells. In the longer timescales, chemically functionalized nanotubes have the ability to reorient vertically to the membrane axis and pierce the membrane. However, the parallel orientation was especially favorable when the carbon nanotubes were modified with positively charged groups and were brought into contact with negatively charged membranes. This is also the case in the present study, where carbon nanotubes are modified with positively charged doxorubicin and interact with negatively charged thiolipid (Fig. 1). Additionally, the parallel orientation of nanotubes with respect to the lipid membrane would result in relatively small increase in the area per molecule in the organized monolayer as observed in Langmuir studies (Table 2). A scheme explaining the mechanisms of interaction of nanotubes and lipids is shown in Fig. 8. Different possibilities of the orientation of nanotubes in the layer: parallel with respect to the lipid monolayer, formation of bigger aggregates as well as insertion of the nanotubes into the thiolipid layer reflecting the interactions with both hydrophobic chains and polar region are presented.

The most important changes caused by the presence of nanotubes concern the compression modulus and its maximum value. The reciprocal of compression modulus is defined as (Gaines 1966)

$$ {Cs}^{-1}=-A\frac{d\pi}{d A} $$

where *A* is area per molecule, and π is surface pressure. This parameter provides the information on the state, in which the monolayer is at a given surface pressure. The maximum value of compression modulus changes significantly in the presence of nanotubes in the mixed monolayers (Table 2). The decrease from the value of approximately 400 mN/m obtained for monocomponent DPPTE monolayers corresponding to the solid state of the monolayer to the values of approximately 160 mN/m shows that the presence of drug carriers leads to the formation of a layer, which is now in the liquid-condensed state (Harkins 1952). It suggests that the presence of carbon nanotubes changes the organization of DPPTE molecules and prevents from their tighter packing at the air–water interface and thus causing the monolayer to become more fluid. Interestingly, the decrease in the maximum value of compression modulus does not depend on the SWCNT-DOx content in the mixed layer and remains at the similar level for all the investigated DPPTE/SWCN-DOx ratios with the prevailing amount of the nanotubes (inset in Fig. 6a and Table 2).

Different situation is observed when mixed DPPTE/SWCNT-DOx layers contain the prevailing amount of the thiolipid with respect to nanotubes (Fig. 6b). In this case, the characteristic parameters of the monolayer such as the area per molecule in the organized monolayer, area per molecule corresponding to the collapse, and collapse pressure do not change in the presence of relatively small amounts of carbon nanotubes (Table 2). Additionally, the reciprocal of compression modulus does not change either (inset in Fig. 6b) and its maximum value remains at the similar level compared to the value observed for monocomponent DPPTE monolayers (Table 2). It may be therefore supposed that relatively small amounts of carbon nanotubes can be accommodated in the mixed monolayer and somehow they do not influence monolayer organization and structure.

### Electrochemical comparison of SWCNT-DOX end and side adducts

**Fig. 9:**
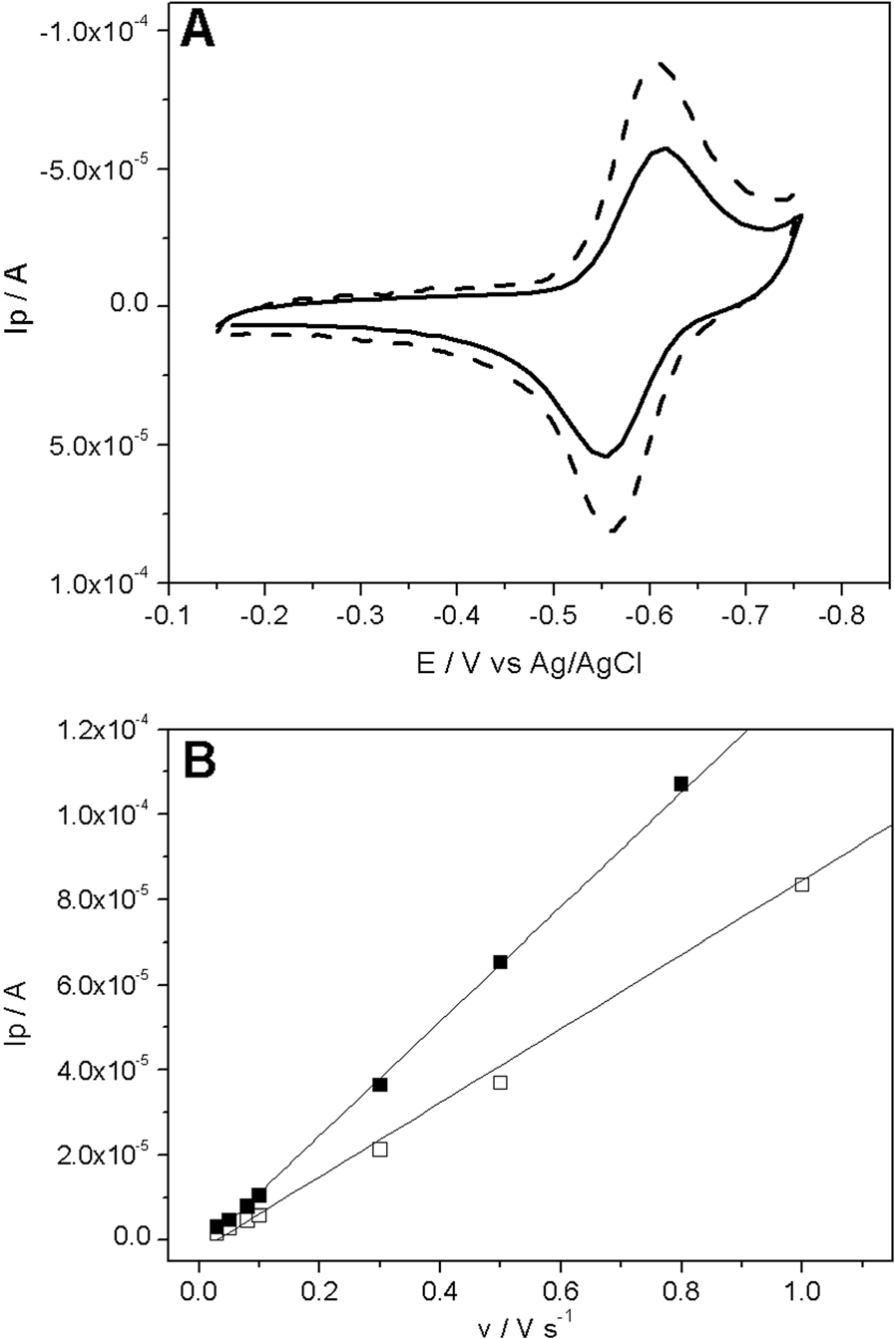
**a** Cyclic voltammograms obtained for supported mixed DPPTE/SWCNT-DOx (1:10 *w*/*w*) monolayers for end modification (solid) and side modification (doted). Scan rate 0.5 V/s. **b** Dependence of peak current vs. scan rate for end modification (open squares) and side modification (filled squares)

Prior to the electrochemical characterization, the mixed DPPTE/SWCNT-DOx monolayers were transferred onto gold electrodes by means of Langmuir–Blodgett technique (the “Monolayer deposition on solid support and electrochemical experiments” section). Then, electrochemical techniques have been employed in order to compare the two types of modification of the nanotubes. It was possible due to the electrochemical activity of doxorubicin attached to carbon nanotubes. Cyclic voltammetry was performed for mixed layers of DPPTE/SWCNT-DOx (1:10 *w*/*w*) for both end and side modifications of the nanotubes (Fig. 9a). The oxidation-reduction peaks correspond to the electrode process of the quinone-hydroquinone group present in the doxorubicin molecule (Komorsky-Lovrić 2006) and prove the presence of the drug at the electrode surface. However, electrochemistry also gives the possibility to obtain additional information on the nature of the attachment of the drug to the nanotubes. Since the dependence of peak current on scan rate is linear, it proves the adsorptive character of the electrode process of doxorubicin (Saveant 2006), and therefore, it may be stated that the prepared carbon nanotube–drug composite remains attached to the electrode surface (Fig. 9b). Linear dependence of peak current on the scan rate was observed for both end and side modification of nanotubes with doxorubicin, which were used to prepare mixed layers. Additionally, the multiple cyclic voltammograms recorded for the electrodes modified with the mixed DPPTE/SWCNT-DOx (1:10 *w*/*w*) monolayers showed that there is no significant difference in peak current observed with the increasing number of scans recorded (Fig. 1s). Therefore, it proves that the electroactive drug is not weakly linked onto the electrode surface but it is covalently bonded to the supported nanotubes and is not released to the supporting electrolyte, which would result in the decrease in peak current with the increasing number of scans.

The two types of modification of nanotubes used in this study, namely, covalently modified nanotubes either at the end or side of nanotubes, were also compared by means of electrochemical methods. Cyclic voltammograms obtained for electrodes modified with mixed layers prepared using these two types of carbon nanotubes were recorded (Fig. 9a). As it can be observed, the peak currents corresponding to the redox reaction of doxorubicin are larger for the electrode modified with the mixed layer containing nanotubes with drug attached to the sides of the nanotubes. This seems reasonable since the side modification allows for more modification sites. The surface concentration of doxorubicin attached to the nanotubes and present at the electrode surface can be estimated using the following equation:

$$ \Gamma =\mathrm{Q}/\mathrm{nFA} $$

where Γ is surface concentration (mol/cm^2^), Q is charge under cathodic peak (C), *n* is number of electrons, *F* is Faraday constant, and *A* is electrode area (cm^2^). The surface concentration calculated based on the cyclic voltammograms presented in Fig. 9 are equal to 0.66·10^−10^ and 1.08·10^−10^ mol/cm^2^ for end and side modification, respectively. The value of surface concentration obtained for the side modification may be compared to the value of 3.6·10^−10^ mol/cm^2^ reported previously for mixed MWCNT-AQ/octadecanol monolayer (10:1 *w*/*w*), in which the multi-walled carbon nanotubes were modified on the sides with anthraquinone (Sadowska et al. 2009). Other published data on carbon nanotubes derivatized with anthraquinone report the surface concentration of 2.04·10^−10^ mol cm^−2^ (Banks et al. 2005). Additionally, it should be noticed that the application of the carbon nanotubes modified with the drug at the ends gives the values of surface concentration, which constitute 60% of the value of surface concentration obtained for the side modification. Results leading to the conclusion that side modification is more efficient compared to the end modification were also obtained by Sadowska et al. (2010), who compared SWCNTs modified either on sidewalls or at the ends with ABTS, a redox mediator used for the construction of electrodes for biocatalytic oxygen reduction by laccase. It also proves that side modification is more efficient and more promising as far as the delivery of doxorubicin by means of carbon nanotubes is concerned.

## Conclusions

Carbon nanotubes-doxorubicin adducts were synthesized, characterized, and studied with respect to their influence as potential drug carriers on the model biological membranes composed of a negatively charged thiolipid, DPPTE. The anticancer drug was covalently attached either on the side or at the end (and defect sites) of the nanotubes. The obtained adducts were first characterized by thermogravimetry and spectroscopic techniques in order to confirm the covalent attachment of the drug. SWCNT-DOx were added to DPPTE to form mixed monolayers with different weight ratio of the components and studied by Langmuir technique. The model membranes were found to change their properties under the influence of carbon nanotubes modified with the anticancer drug. Relatively small amount of nanotubes did not affect the isotherm shape and properties, which implies that small amounts of nanotubes may be accommodated within the mixed layer. The introduction of larger amounts of carbon nanotubes into the monolayer results in the increase in the average area per molecule in the monolayer with considerable impact of nanotubes observed in the beginning of the compression of the monolayer when the membranes is less organized. Additionally, the presence of drug carriers makes the model membranes less condensed. The changes induced in the membrane structure, e.g., fluidization and changes in the organization of the phospholipid molecules, may imply that the drug carrier can in this way strongly interact with the membrane or possibly cross this barrier.

Mixed layers of DPPTE/SWCNT-DOx were also transferred onto gold electrodes by means of Langmuir–Blodgett method, and the amount of the drug present in the transferred membrane was determined by cyclic voltammetry. The linear relationship between scan rate and peak current and reproducibility of the voltammograms confirm that SWCNT-doxorubicin remain in the layer at the electrode surface, and the composite layer is stable in time. Additionally, higher values of peak charges proved that surface concentration of the drug achieved for the side modification is larger than for the end modification making the former more favorable for further studies as drug delivery systems.

## Electronic supplementary material

ESM 1 (DOCX 24 kb)
