# Supplementary material for: Synthesis, characterization, and interactions of single-walled carbon nanotubes modified with doxorubicin with Langmuir–Blodgett biomimetic membranes
Source: J Nanopart Res. 2018 May 12;20(5):143. doi: 10.1007/s11051-018-4239-x (PMC5949139; doi:10.1007/s11051-018-4239-x)
Supplement: Supplementary file 1 — (DOCX 24 kb) [file 11051_2018_4239_MOESM1_ESM.docx]

**SUPPORTING INFORMATION**

**Synthesis, characterization and interactions of single-walled carbon nanotubes modified with doxorubicin with Langmuir-Blodgett biomimetic membranes.**

Dorota Matyszewska^1^, Ewelina Napora^2^, Kamila Żelechowska^3^, Jan F.Biernat^4^, Renata Bilewicz^2*^

*^1^Faculty of Chemistry, Biological and Chemical Research Centre, University of Warsaw,*

*ul. Żwirki i Wigury 101, 02-089 Warsaw, Poland*

*^2^Faculty of Chemistry, University of Warsaw, ul. Pasteura 1, 02-093 Warsaw, Poland*

*^3^Faculty of Applied Physics and Mathematics, Gdansk University of Technology, Narutowicza 11/12, 80-233 Gdansk, Poland*

*^4^Chemical Faculty, Gdansk University of Technology, ul. Narutowicza 11/12, 80-233 Gdansk, Poland*

Figure 1S. Multiple cyclic voltammograms obtained for supported mixed DPPTE:SWCNTs-DOx (1:10 w/w; end modification) monolayer: 2 scan (black), 5 scan (red), 10 scan (green), 15 scan (blue), 20 scan (cyan), 25 scan (magenta), 30 scan (yellow). Scan rate 0.1 V/s.
